# How Community Nurses Modify Relational, Social, and Institutional Environments to Enable Participation in a Rural Japanese Village: A Secondary Qualitative Analysis

**DOI:** 10.3390/healthcare14111527

**Published:** 2026-06-01

**Authors:** Ryuichi Ohta, Akiko Yata, Chiaki Sano

**Affiliations:** 1 Department of Community Care, Unnan City Hospital, Unnan 699-1221, Shimane, Japan; 2 Community Nurse Company, Unnan 699-1332, Shimane, Japan; yataakiko0425@gmail.com; 3 Community Medicine Management, Faculty of Medicine, Shimane University, Izumo City 693-8501, Shimane, Japan

**Keywords:** community health nursing, social participation, rehabilitation, social environment, qualitative research

## Abstract

**Background/Objectives**: Participation is a central outcome of rehabilitation and is influenced by environmental factors, as described in the International Classification of Functioning, Disability and Health (ICF). However, environmental factors are often conceptualized as static conditions, and the mechanisms through which healthcare professionals actively modify environments to enable participation remain insufficiently understood. This study aimed to examine how community nurses modify environmental factors to enable participation in community settings. **Methods**: This qualitative study conducted a secondary analysis of 10 available monthly Community Nurse activity logs and reflective practice records from January, February, April, and June to December 2025 in a rural community. Thematic analysis was performed to identify patterns in how community nurses modified environmental contexts to enable participation. Analysis was informed by the environmental factors component of the ICF framework. **Results**: Four interrelated environmental modification processes were identified: creating participation-enabling social environments; strengthening relational environments through trust and validation; restructuring social roles and participation expectations; and bridging digital, structural, and institutional barriers to participation. Community nurses facilitated recreational and social activities, invited residents who were not yet participating in ongoing activities, coordinated with local organizations, and supported reciprocal resource sharing. These actions reconstructed participation environments, enabling residents to engage in meaningful social interaction, assume active roles, and sustain community engagement. **Conclusions**: Community nurses enabled participation by actively modifying environmental factors across physical, relational, and institutional domains. Environmental modification functioned as a key mechanism enabling participation in community-based rehabilitation. These findings operationalize the environmental factors component of the ICF framework and highlight the importance of environmental facilitation in participation-oriented rehabilitation.

## 1. Introduction

Participation, defined in the International Classification of Functioning, Disability and Health (ICF) as involvement in life situations, is a key outcome of rehabilitation and an important determinant of health and well-being [1]. Participation is associated with improved social integration, psychological well-being, and reduced risks of functional decline among older adults and individuals with chronic health conditions [2,3]. Consequently, rehabilitation has increasingly shifted toward participation-oriented approaches that support engagement in meaningful social roles and community life [4].

The ICF framework conceptualizes participation as emerging from dynamic interactions among health, personal, and environmental factors [5]. Environmental factors include the physical, social, relational, and institutional contexts in which individuals live, such as support networks, attitudes, services, and community infrastructure [6,7]. These environmental contexts can either facilitate or hinder participation. Supportive relationships, accessible community spaces, and inclusive social environments may enable individuals to engage in meaningful activities. In contrast, social isolation, fragmented services, and limited access to community resources may restrict participation [8,9,10]. Accordingly, modifying environmental conditions has become an important strategy for participation-oriented rehabilitation.

Despite the recognized importance of environmental factors, their operationalization in rehabilitation practice remains insufficiently understood. Many previous studies have treated environmental factors as static contextual characteristics, such as physical accessibility or service availability [11]. However, environmental contexts are dynamic and continuously shaped through social interactions and facilitative practices within communities. There is limited empirical research examining how environmental conditions are actively modified to enable participation in real-world community settings. Consequently, the mechanisms through which environmental contexts are reshaped to support participation remain unclear.

Community Nurses (CNs), who are embedded within community environments and engage in sustained relational interactions with residents, may play a critical role in modifying environmental conditions [12]. Unlike conventional healthcare providers who primarily focus on clinical care, CNs operate within social and community contexts by facilitating relationships, connecting individuals with resources, and fostering inclusive community environments as public health nurses [13]. Through these practices, CNs may reshape environmental conditions by strengthening social support networks, modifying relational dynamics, and bridging structural barriers that limit participation.

Although previous research has highlighted the role of CNs in facilitating participation, the specific mechanisms by which CNs modify environmental factors to enable participation remain poorly understood [12]. Previous research has demonstrated that community nurses reconstruct participation through relational facilitation in community settings [14]. However, the mechanisms initiating participation remain unclear. There is a lack of empirical research examining how environmental contexts are actively reconstructed through community-based rehabilitation practices. Understanding these mechanisms is essential for advancing participation-oriented rehabilitation and for operationalizing the Environmental factors component of the ICF framework in real-world practice.

Therefore, this study aimed to examine how CNs modified relational, social, digital, and institutional environmental factors to enable social participation among community-dwelling residents in a rural Japanese village, using the environmental factors component of the International Classification of Functioning, Disability and Health framework as an analytical lens. In this study, social participation referred to residents’ engagement in everyday community life, including informal conversations, community café activities, recreational activities, peer support, cultural activities, and connections with local organizations. The research question guiding this study was: How do Community Nurses actively modify relational, social, digital, and institutional environmental conditions to enable social participation among community-dwelling residents in a rural Japanese village?

## 2. Materials and Methods

### 2.1. Study Design

This study employed a retrospective qualitative design using a secondary analysis of Community Nurses’ (CNs’) routine practice records to examine how CNs modified environmental factors to enable participation in community settings. The analysis was based on the monthly Hyakuwaq Reports and associated reflective descriptions documented as part of ongoing CN practice in Sarabetsu Village. The shared dataset comprised 10 monthly reports from January, February, April, and June through December 2025. Reports from March and May were not included because they were unavailable in the shared anonymized dataset used for this secondary analysis.

This study was conducted as a secondary qualitative analysis of a broader dataset of Community Nurse practice. A previous related study using the same broader dataset examined how social participation was reconstructed through Community Nurse practice [14]. However, the present study had a distinct research question, theoretical framework, unit of analysis, coding focus, and thematic structure.

The previous study focused on the process through which social participation emerged and was reconstructed in community settings. In contrast, the present study specifically focused on how Community Nurses modified environmental conditions that made participation possible. The analysis was informed by the environmental factors component of the International Classification of Functioning, Disability and Health framework.

To maintain clear analytical boundaries, we conducted a separate data extraction and coding process for the present study. We extracted only those segments that described Community Nurse actions that modified relational, social, digital, physical, psychological, or institutional environmental conditions affecting residents’ participation. Segments that described participation experiences without identifiable environmental modification were not included in the present analysis. The codes and themes in this study were newly generated and were not reused from the previous study (Table 1).

Therefore, the present study does not divide the same analysis into multiple publications. Rather, it provides a distinct analysis of environmental modification as a mechanism enabling participation in rural community settings.

### 2.2. Study Setting

This study was conducted in Sarabetsu Village, a rural agricultural municipality in Hokkaido, Japan. According to the 2020 national census, the total population of Sarabetsu Village was 3080, and the population was 3084 as of the end of December 2024, according to the Basic Resident Register. The proportion of residents aged 65 years or older was 31.5% in 2020, higher than the national average of 28.8%, and is projected to increase to approximately 40.9% by 2050.

Sarabetsu Village is characterized by a small and aging population, dispersed residential areas, an agricultural economy, and rural living conditions in northern Japan. In such contexts, everyday social participation may be affected by population aging, limited public transportation, reduced opportunities for informal social interaction, digital exclusion, and potential social isolation among older residents. These contextual features made Sarabetsu Village an appropriate setting for examining how Community Nurses modified relational, social, digital, and institutional environments to enable participation among community-dwelling residents.

Three CNs were embedded in the community and engaged with residents across a wide range of everyday settings. In the shared records, CN practices took place in community hubs such as Midori no Ie, the onsen lobby, the welfare center, the village office, and residents’ homes. CN activities extended beyond conventional clinical care and included inviting residents to community events, facilitating social interaction, supporting digital inclusion, connecting residents with local resources, and helping residents re-enter or sustain participation in valued activities. These features indicate that CNs worked not only as care providers but also as relational and environmental facilitators within the community.

### 2.3. Community Nurse Practice Model

In Sarabetsu Village, the Community Nurse practice model was implemented by three Community Nurses who were embedded in the community and engaged with residents in everyday settings. The target population was community-dwelling residents, particularly older adults, those at risk of social isolation, and individuals who had difficulty accessing or continuing to participate in community activities. The model was not limited to conventional facility-based or home-based clinical nursing care. Rather, it focused on identifying barriers to participation in everyday life and modifying community environments to make participation more feasible.

Community Nurse activities were conducted across multiple community settings, including Midori no Ie, the onsen lobby, the welfare center, the village office, local cafés, community gathering spaces, and residents’ homes. CNs engaged with residents through informal conversations, home visits, community café activities, recreational events, smartphone support sessions, book lending, haiku gatherings, event invitations, and follow-up interactions in public and private spaces.

The main functions of the Community Nurse model included: identifying residents who were socially isolated or hesitant to participate; inviting residents to community activities; creating low-threshold participation spaces; facilitating relationships among residents; validating residents’ interests and life experiences; supporting digital inclusion through smartphone-related assistance; connecting residents with local resources; coordinating with community organizations and administrative structures; and encouraging residents to assume active roles within community activities.

Thus, the Community Nurse practice model examined in this study comprises four interrelated components: community embeddedness, relational facilitation, resource connection, and environmental modification. These components provided the practical basis for analyzing how CNs modified relational, social, digital, physical, psychological, and institutional environmental conditions to enable residents’ participation in community life.

### 2.4. Data Source and Data Collection

The primary data source consisted of available routine CN practice records documented in the monthly Hyakuwaq Reports in 2025, together with reflective descriptions of CN-resident interactions and community activities. The shared dataset comprised 10 monthly reports from January, February, April, and June through December 2025. Reports from March and May were not included because they were unavailable in the shared anonymized dataset used for this secondary analysis. Therefore, the present study analyzed only the records that had been anonymized and made available for research use.

These records included narratives of resident encounters, event facilitation, home visits, opportunistic conversations in public spaces, practical support for participation, and reflections on the relational dynamics and contextual conditions that shaped participation. The records captured naturally occurring community practices, including smartphone support sessions, haiku gatherings, game-based social events, community café activities, book lending, event invitations, and follow-up interactions in homes and public spaces.

For this study, we extracted data segments in which CNs actively modified environmental contexts in ways that could influence residents’ social participation. Environmental modification was operationally defined as CN actions that altered relational, social, psychological, digital, physical, or structural conditions affecting residents’ ability to engage in everyday community life. Segments were included when they described CN actions that modified or attempted to modify environmental conditions linked to actual or potential participation. Segments were excluded when they described only administrative information, general community events without identifiable CN involvement, or clinical support without clear environmental modification. These extracted segments formed the analytic dataset for thematic analysis.

### 2.5. Data Segment Extraction

For this study, relevant data segments were extracted from the available monthly Hyakuwaq Reports and associated reflective descriptions. The first author initially reviewed all available reports and identified segments in which CNs actively modified, or attempted to modify, environmental contexts that could influence residents’ social participation.

Segments were included in the analytic dataset when they met the following criteria: (1) the segment described a CN action or CN-facilitated activity; (2) the action modified or attempted to modify a relational, social, psychological, digital, physical, or institutional environmental condition; and (3) the modification was linked to actual or potential resident participation in everyday community life.

Segments were excluded when they described only administrative information, general community events without identifiable CN involvement, routine clinical support without clear environmental modification, or participation experiences without identifiable modification of environmental conditions.

After the initial extraction, the second author reviewed the extracted segments to assess their relevance to the study aim and inclusion criteria. Any disagreements or uncertainties regarding segment selection were discussed between the first and second authors until agreement was reached. This process provided investigator triangulation at the data selection stage and helped ensure that the analytic dataset was consistent with the research question and theoretical framework.

### 2.6. Analytical Framework

This study was informed by the participation component of the International Classification of Functioning, Disability and Health (ICF), with particular attention to environmental factors influencing participation [1]. Within the ICF, environmental factors include the physical, social, attitudinal, and institutional conditions in which people live and conduct their lives. In this study, environmental factors were operationally defined as modifiable contextual conditions affecting participation, including relational environments such as interpersonal support and familiarity, social environments such as accessible gathering opportunities, structural environments such as links to services and community systems, digital environments such as access to smartphone-mediated communication, and psychological environments such as perceived safety, welcome, and belonging. Psychological accessibility was inferred from residents’ repeated attendance, willingness to ask questions openly, and expressions of wanting to return with others.

The analysis, therefore, focused on how CN practices actively reshaped these conditions and how such modifications supported participation in everyday community contexts. This framework enabled us to conceptualize environmental factors not as static background conditions but as dynamic, modifiable mechanisms that emerge through practice. 

### 2.7. Qualitative Data Analysis

Data were analyzed using reflexive thematic analysis, guided by Braun and Clarke’s six-phase framework [15,16,17]. Reflexive thematic analysis was selected because it enables an interpretive examination of patterned meanings across qualitative data while allowing iterative movement between the dataset, codes, candidate themes, and the developing conceptual account. This approach was appropriate for the present study because the aim was not to quantify predefined categories, but to interpret how Community Nurses modified environmental conditions to enable social participation in everyday rural community contexts.

RO extracted 82 data segments from the available monthly Hyakuwaq Reports and associated reflective descriptions. These segments described Community Nurse actions or CN-facilitated activities that modified, or attempted to modify, relational, social, psychological, digital, physical, or institutional environmental conditions related to residents’ participation.

RO then generated 46 initial codes inductively from these extracted segments. The initial codes captured concrete environmental modification processes, such as creating drop-in spaces, personally inviting residents, facilitating resident-to-resident teaching, supporting smartphone use, adjusting activity spaces, linking residents with community events, coordinating with local organizations, and enabling residents to contribute food, equipment, knowledge, or practical support.

These initial codes were compared and grouped into 12 candidate subthemes according to conceptual similarity and functional relationships. Candidate subthemes were reviewed in relation to the coded data, the full dataset, and the environmental factors component of the ICF framework. Through this iterative process, the 12 candidate subthemes were refined into four final themes: creating participation-enabling social environments; strengthening relational environments through trust and validation; restructuring social roles and participation expectations; and bridging digital, structural, and institutional barriers to participation.

During theme refinement, some preliminary categories were merged because they overlapped conceptually. For example, codes related to informal invitation, drop-in participation, flexible attendance, and observation before participation were grouped into the subtheme “lowering entry barriers”, which contributed to the final theme “creating participation-enabling social environments.” Similarly, codes related to peer teaching, resident-to-resident support, sharing skills, and explaining digital tools were grouped into the subtheme “enabling reciprocal support”, which contributed to the final theme “restructuring social roles and participation expectations”.

AY reviewed the coding structure, candidate subthemes, theme labels, and representative data extracts after RO completed the initial coding and preliminary theme development. Interpretive differences were discussed through repeated comparison with the original data segments. During these discussions, RO and AY examined whether each code and subtheme accurately reflected the data, whether candidate themes were internally coherent, whether theme boundaries were sufficiently distinct, and whether the interpretation was consistent with the research question and the environmental factors component of the ICF framework.

Differences in interpretation were resolved by refining code labels, revising subtheme definitions, merging conceptually overlapping categories, or excluding segments that did not clearly describe environmental modification. For example, segments describing residents’ general enjoyment of community activities were not included in the final themes unless they also showed how CNs modified relational, social, digital, physical, psychological, or institutional conditions. The final thematic structure was determined when RO and AY agreed that the themes were sufficiently grounded in the data, conceptually distinct, and theoretically aligned with the study aim.

To improve analytical transparency, Table 2 shows the relationship among representative initial codes, candidate subthemes, final themes, and the number of coded segments contributing to each final theme.

### 2.8. Rigor and Trustworthiness

To enhance rigor and trustworthiness, we considered the four criteria proposed by Lincoln and Guba: credibility, dependability, confirmability, and transferability [18]. These criteria were used to guide the design, analysis, and reporting of this qualitative secondary analysis.

Credibility was strengthened through repeated engagement with the available monthly Hyakuwaq Reports and associated reflective descriptions. RO repeatedly read the 10 available monthly reports to become familiar with the dataset and identify recurring patterns of environmental modification. Investigator triangulation was also used. RO conducted the initial segment extraction, coding, and candidate theme development. At the same time, AY reviewed the coding structure, candidate subthemes, theme labels, and representative data extracts from the perspective of Community Nurse practice and organizational development. Interpretive differences were discussed by returning to the original data segments and examining whether the interpretations were sufficiently supported by the data and aligned with the research question.

Dependability was supported by maintaining an audit trail throughout the analytic process. This included records of segment extraction, inclusion, and exclusion decisions; initial codes; candidate subthemes; theme refinement; merged or discarded preliminary categories; and analytic memos. The analytic pathway from extracted segments to initial codes, candidate subthemes, and final themes was summarized in Table 2 to improve transparency and reproducibility.

Confirmability was enhanced by grounding interpretations in the original data segments and by documenting reflexive analytic memos. RO recorded preliminary interpretations, contextual observations, and decisions made during coding and theme development. AY reviewed representative extracts and questioned whether the interpretations reflected Community Nurse practice and the environmental factors component of the ICF framework. This process helped reduce the influence of a single researcher’s assumptions and ensured that the findings remained grounded in the data.

Transferability was supported by providing a detailed description of the study setting, the Community Nurse practice model, the characteristics of Sarabetsu Village, and the community contexts in which participation support occurred. These included community hubs such as Midori no Ie, the onsen lobby, the welfare center, the village office, local cafés, public spaces, and residents’ homes. By describing the rural, aging, and community-based context in detail, we aimed to enable readers to judge the applicability of the findings to other rural or community-based rehabilitation settings.

### 2.9. Reflexivity

RO is a physician and qualitative researcher with expertise in community-based healthcare and rehabilitation. Although RO was not directly involved in the CN activities described in the dataset, this background influenced the interpretation of participation and environmental facilitation. Reflexive awareness was therefore maintained throughout familiarization, coding, and interpretation. AY has extensive experience in Community Nurse practice and organizational development and contributed contextual and practice-based insight while critically reviewing interpretations. Ongoing reflexive dialogue between the authors supported analytic rigor. 

### 2.10. Ethical Considerations

This study used anonymized routine practice records and reflective descriptions originally documented as part of CN activities in the community. All personal identifiers were removed before analysis. Because the records were retrospective and anonymized, the requirement for individual informed consent was waived. Ethical approval was obtained from the Ethics Committee of Unnan City Hospital, Shimane, Japan (approval number: 2025005).

## 3. Results

### 3.1. Overview of Environmental Modification Processes Facilitated by Community Nurses

Analysis of CN activity logs and reflective practice records identified multiple instances in which CNs actively modified environmental contexts to enable participation among community residents. These environmental modifications occurred across diverse community settings, including community gathering spaces such as “Midori no Ie”, local cafés, public facilities, and residents’ homes, and involved relational, social, and structural environmental changes.

Environmental modification was closely linked to concrete participation events that emerged through CN facilitation. For example, at a community gathering at “Midori no Ie”, CNs facilitated recreational activities such as table tennis and board games. Residents collaboratively prepared the environment: one resident transported a table tennis table in his personal vehicle, while others helped arrange the space. Although the table occupied most of the room, participants adapted the environment and actively engaged in playing together, laughing, observing others, and conversing. This event enabled not only physical engagement but also social interaction, including spontaneous conversations among residents who had not previously interacted.

Similarly, CNs facilitated participation through community café activities. Residents who had previously remained socially isolated were personally invited by CNs to attend café gatherings. One resident, initially hesitant and surprised at being invited, subsequently attended the café and engaged in conversations with other participants while sharing tea and food. During these interactions, participants discussed their daily lives, health concerns, and past experiences, demonstrating meaningful social engagement. In some cases, residents who initially attended as observers gradually became active participants, contributing to conversations and assisting with event preparation.

CNs also facilitated access to books and informal cultural resources. For example, one resident browsed the bookshelves at Midori no Ie, asked whether books could be borrowed, and took home books of interest. One resident expressed interest in reading and later returned to discuss the book’s content with CNs and other participants. This interaction fostered cognitive engagement and social exchange, illustrating participation that extends beyond physical activity.

Environmental modification also enabled reciprocal participation through resource sharing. During community activities, residents voluntarily brought food and shared it with others, assisted with preparation tasks, and contributed equipment. These actions enabled residents to assume active roles within the participation environment, shifting from passive recipients of support to contributors to the shared social context.

These concrete participation events illustrate that environmental modification was not limited to improving physical accessibility but involved dynamic reconstruction of social environments, relational networks, and participation infrastructures. CNs functioned as facilitators, reshaping environmental contexts to enable residents to engage in meaningful life situations, including social interaction, community participation, and role engagement.

These findings demonstrate that CN practice operationalizes environmental factors within the ICF framework by actively modifying environmental conditions, enabling participation to emerge through interaction between individuals and their reconstructed environments. Concrete examples of CN-facilitated environmental modification and participation events are summarized in Table 3.

### 3.2. The Result of Thematic Analysis

Thematic analysis identified four interrelated themes describing how CNs modified environmental factors to enable participation: (1) creating participation-enabling social environments, (2) strengthening relational environments through trust and validation, (3) restructuring social roles and participation expectations, and (4) bridging digital, structural, and institutional barriers to participation. These themes illustrate how environmental factors were actively reconstructed through CN practice. The thematic analysis demonstrated that CN activities modified multiple environmental domains within the ICF framework. The relationship between identified themes and corresponding ICF environmental factors is summarized in Table 4.

### 3.3. Theme 1: Creating Participation-Enabling Social Environments

CNs modified environmental contexts by creating socially and psychologically accessible spaces where residents could enter, observe, stay briefly, or participate without formal obligation. This theme was supported by 24 coded segments. These segments showed that participation was enabled not only by organizing activities but also by making community spaces flexible, familiar, and easy to approach.

A central feature of this theme was the creation of low-threshold participation spaces. CNs used everyday community locations, such as Midori no Ie and the onsen lobby, as open and informal places where residents could join activities at their own pace. For example, during an e-sports activity at Midori no Ie, one resident arrived with a smile and said, “I walked here for the first time”. Although she was initially cautious about overexertion, she later agreed to continue participating, saying, “Since I am here, let’s do it”. This episode illustrates how an accessible and flexible environment encouraged residents to move from tentative attendance to active participation.

CNs also recognized that psychological accessibility depended on where and how encounters occurred. Some residents described Midori no Ie as meaningful but still somewhat difficult to enter, whereas the onsen lobby felt easier for casual interaction. One resident stated, “Midori no Ie is good, but it still feels a little difficult to enter. It is easier to talk when you are at the onsen”. This extract shows that participation-enabling environments were not limited to formally prepared spaces. Rather, CNs modified participation opportunities by positioning themselves in familiar public settings where residents already felt comfortable.

At Midori no Ie, CNs also transformed ordinary space into a participation-ready environment through flexible activity design. During a game-based event, CNs prepared table tennis, mahjong tile-stacking, e-sports, music, and small seasonal incentives, while keeping the space open throughout the day. Residents did not merely attend the event; they helped shape the participation environment. One resident brought decorations, another contributed apples, and others welcomed people who were looking in from the outside. The record described that when someone was looking into the space, residents called out, “Come in!”, indicating that a socially accessible environment was co-created by CNs and residents.

Informal invitations also helped convert everyday movement into participation. For example, when a resident was walking past Midori no Ie, CNs called out to her from the window. She returned and said, “I am glad you called out to me”. Such small acts of recognition lowered psychological barriers and enabled residents to enter a community space without needing a formal reason for participation.

### 3.4. Theme 2: Strengthening Relational Environments Through Trust and Validation

CNs actively modified relational environments by fostering trust, validating residents’ experiences, and strengthening interpersonal connections. This theme was supported by **20 coded segments**. These segments showed that participation became more feasible when residents felt recognized, remembered, listened to, and valued within everyday community interactions.

A central feature of this theme was the validation of residents’ life histories and personal interests. CNs not only invited residents to activities; they also listened to their memories, skills, concerns, and aspirations in ways that affirmed residents’ identities. For example, when one resident spoke about having self-published a record during high school, the CN responded with interest and asked to hear more about his music. The resident then said, “If I have a guitar again, I will perform a song”. This interaction illustrates how recognition of a resident’s past experiences and abilities transformed an ordinary conversation into a relational environment that supported future participation.

Relational environments were also strengthened when CNs created spaces where residents could safely share personal memories and emotions. During a haiku gathering, one resident shared deeply personal memories, including an episode from the day before his divorce and a memory related to his uncle’s funeral. Another resident responded with empathy, saying that hearing the story brought tears to her eyes, and offered thoughtful advice about the wording of his haiku. This exchange shows that CN-facilitated activities did not merely provide recreation; they created relational spaces where residents could be heard, emotionally supported, and connected through shared reflection.

Personal recognition also reduced psychological barriers to participation. For example, when a resident was walking past Midori no Ie, CNs called out to her from the window. She returned and said, “I am glad you called out to me”. This short exchange demonstrates how being recognized by name and personally invited could transform a passing encounter into participation. Such relational accessibility made community spaces feel more welcoming and less formal.

CNs also facilitated connections among residents by identifying shared interests and encouraging interaction. In several activities, residents introduced others to community events, invited acquaintances to join, exchanged stories, and supported each other’s engagement. For instance, in the Christmas gathering, residents interacted through books, ukulele, games, and shared conversation. One resident expressed joy when another arrived, saying, “I was waiting for you”. These interactions suggest that CNs helped create relational environments in which residents were not only connected to CNs but also increasingly connected to one another.

### 3.5. Theme 3: Restructuring Social Roles and Participation Expectations

CNs modified participation environments by enabling residents to move from being recipients of support to becoming contributors, supporters, and co-creators of community activities. This theme was supported by 21 coded segments. These segments showed that participation was not limited to attending activities; rather, residents gradually assumed active roles by teaching others, bringing resources, preparing spaces, sharing knowledge, and supporting the participation of other residents.

This role restructuring was most clearly observed during the smartphone support session at Midori no Ie. The session was initially organized in response to residents’ requests for smartphone support. However, the participation environment became reciprocal as residents and community members naturally assumed teaching roles. The report stated that several participants “naturally became the ones teaching others”, and residents helped one another with LINE registration, QR-code scanning, Wi-Fi connection, and smartphone functions. For example, one resident who already knew how to register LINE friends using QR codes was asked to support another participant and willingly took on the role of explaining the process. This episode shows that CNs did not simply provide one-way support; they created conditions in which residents’ existing knowledge could become a community resource.

Residents also contributed to participation environments through practical support and preparation. During a game-based event at Midori no Ie, one resident helped transport a table tennis table using his truck, while others brought apples, prepared coffee, or contributed seasonal decorations. One resident arrived saying, “I came to make coffee today”, and prepared coffee according to the other participants’ preferences. Another resident brought apples from home and offered them for everyone to share. These actions show that residents were not positioned as passive attendees but as people who could sustain and enrich the participation environment.

Role restructuring also occurred when residents supported activity management and welcomed others into the space. During the same event, residents called out to a person looking in from outside and invited the person to enter. In another community café activity, a resident helped carry equipment and later brought food for high school participants, showing concern for whether they had eaten breakfast. These actions demonstrate how residents assumed informal host, supporter, and caregiver roles within community activities.

CNs further enabled residents to share personal skills and knowledge. In the Christmas gathering, one resident sent various “spot-the-difference” materials by LINE in advance and later guided others through the activity. Another resident shared knowledge about ukulele, guitar, and park golf rules. Such interactions created opportunities for residents to be recognized not only as participants but also as people with expertise, experience, and resources that could benefit others.

### 3.6. Theme 4: Bridging Digital, Structural, and Institutional Barriers to Participation

CNs modified participation environments by bridging digital, structural, and institutional barriers that limited residents’ access to community resources, information, and participation opportunities. This theme was supported by **17 coded segments**. These segments showed that participation was influenced not only by residents’ motivation or relationships but also by practical access to digital tools, community information, transportation, public spaces, and local administrative systems.

A major component of this theme was digital inclusion. CNs supported residents in using smartphones and communication applications that were increasingly necessary for maintaining social connections and accessing community information. During a smartphone support session at Midori no Ie, the activity was organized in response to residents’ expressed needs, including one resident’s wish to “communicate with everyone through LINE” and another resident’s request for a smartphone class for older residents. Participants practiced LINE registration, QR code scanning, Wi-Fi connection, voice input, smartphone photography, and app-based information access. One resident also wanted to learn how to book airline tickets using a smartphone, while another expressed interest in using Zoom for future training. These examples show that digital support was not merely technical assistance; it modified an environmental barrier that affected residents’ ability to communicate, learn, travel, and remain connected.

CNs also bridged access to community information and local participation opportunities. For example, during a consultation about sharing a Google calendar with her husband, one resident also discussed digital community stamps, walking events, music events, and local flyers. The CN suggested posting event information at Midori no Ie, thereby linking individual digital support with wider community participation. This episode illustrates how CNs connected residents’ personal needs with community-level opportunities and information flows.

Structural barriers were also identified in relation to residents who had difficulty leaving home, remembering schedules, or connecting with existing community services. In one report, a local welfare worker described concerns about residents who were unable to leave their homes, residents in supported housing who remained only in shared rooms, and older adults who could not reliably attend community programs because they forgot schedules. These examples show that participation barriers were not only interpersonal but also embedded in everyday routines, service structures, memory difficulties, mobility limitations, and gaps between residents and available resources. CNs’ role was to identify these gaps and consider how their involvement could broaden the range of community-based support.

Institutional bridging was also evident in CNs’ coordination with local administrative structures. For example, one report documented that approval for distributing flyers for the next café event had been obtained from the village office. Although this may appear to be a minor administrative action, it enabled community information to circulate through legitimate local channels and supported residents’ access to future participation opportunities. This demonstrates that CN-facilitated participation depended not only on face-to-face interaction but also on coordination with municipal and organizational systems.

### 3.7. Conceptual Synthesis: Environmental Modification as a Mechanism Enabling Participation

Across the four themes, CNs functioned as environmental modifiers, actively reshaping the conditions that enabled residents’ participation. The findings suggest that environmental factors were not merely static background conditions surrounding residents’ lives. Rather, they were dynamic, modifiable mechanisms that could be changed through CNs’ relational, social, digital, and institutional practices.

Environmental modification occurred across four interrelated dimensions. First, CNs created participation-enabling social environments by lowering entry barriers, preparing flexible community spaces, and enabling residents to join activities without formal obligation. Second, CNs strengthened relational environments through trust and validation by recognizing residents’ life histories, interests, and identities, thereby reducing psychological barriers to engagement. Third, CNs restructured social roles and participation expectations by enabling residents to move from being recipients of support to becoming contributors, peer supporters, and co-creators of community activities. Fourth, CNs bridged digital, structural, and institutional barriers by supporting access to smartphones, community information, local organizations, and municipal systems.

Together, these processes show how CNs operationalized the environmental factors component of the ICF framework in everyday rural community practice. Participation was enabled not only by improving individual capacity or motivation, but also by modifying the environments in which residents encountered others, accessed information, assumed meaningful roles, and connected with community resources. In this sense, environmental modification functioned as a key mechanism through which social participation became more feasible, meaningful, and sustainable in a rural community-based rehabilitation context.

**Figure 1:**
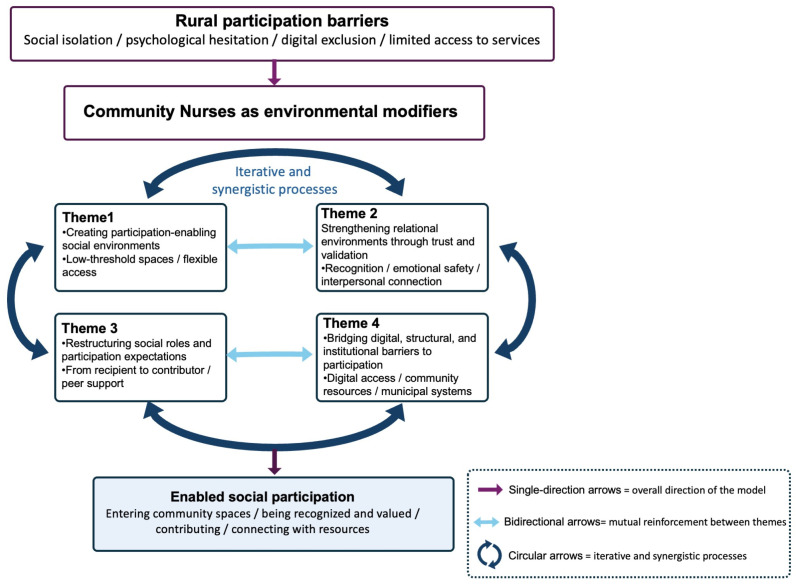
An iterative model of environmental modification by Community Nurses to enable social participation.

The relationships among the four themes and their collective contribution to enabling participation are illustrated in Figure 1.

This figure illustrates how Community Nurses modified environmental conditions to enable social participation in a rural community. Rural participation barriers, including social isolation, psychological hesitation, digital exclusion, and limited access to services, formed the contextual conditions that restricted residents’ participation. Community Nurses functioned as environmental modifiers by engaging in four interrelated processes: creating participation-enabling social environments; strengthening relational environments through trust and validation; restructuring social roles and participation expectations; and bridging digital, structural, and institutional barriers to participation.

The single-direction arrows indicate the overall pathway from rural participation barriers to enabled social participation. The bidirectional arrows among the four themes indicate mutual reinforcement among environmental modification processes. The circular structure indicates that these processes are iterative rather than sequential. Together, the four processes synergistically reconstructed participation-enabling environments by enabling residents to enter community spaces, feel recognized and valued, assume meaningful roles, and connect with community resources and institutional systems.

## 4. Discussion

### 4.1. Summary of the Study

This study examined how Community Nurses modified environmental conditions to enable social participation among community-dwelling residents in a rural Japanese village. This analysis represents one component of a broader qualitative research project examining Community Nurse practices that facilitate participation in rural communities. Using thematic analysis of CN activity logs and reflective practice records, we identified four interrelated environmental modification processes: creating participation-enabling social environments; strengthening relational environments through trust and validation; restructuring social roles and participation expectations; and bridging digital, structural, and institutional barriers to participation.

These findings show that CNs enabled participation not only by supporting individuals but also by modifying the environments in which participation occurred. This interpretation is consistent with the International Classification of Functioning, Disability and Health, which conceptualizes functioning and disability as occurring within a context and includes environmental factors as an essential component of participation and functioning [1]. CNs lowered entry barriers to community spaces, fostered relational trust, enabled residents to assume contributor roles, and connected residents with digital tools, community resources, and local administrative systems. Through these processes, CNs helped residents engage in meaningful life situations, including informal social interaction, reciprocal support, role participation, and community engagement.

The findings also resonate with community-based rehabilitation principles. The WHO Community-Based Rehabilitation guidelines conceptualize CBR as a strategy for community-based development involving people with disabilities and emphasize inclusion, quality of life, participation, and empowerment [19]. In this study, CNs enacted these principles by modifying relational, social, digital, and institutional environments that shaped residents’ opportunities for participation. This is also consistent with previous rehabilitation research showing that environmental barriers and supports influence everyday participation across immediate, community, social, and societal levels [20]. Therefore, CNs may contribute to community-based rehabilitation by reconstructing participation-enabling environments in rural communities.

### 4.2. Comparison with Other Studies

The findings of this study extend the participation-oriented rehabilitation literature by showing how environmental factors can be actively modified in everyday rural community practice. Rather than viewing participation primarily as an individual functional outcome, our findings support an ecological understanding of participation, in which engagement in life situations emerges through interactions among individuals, interpersonal relationships, community resources, digital tools, physical spaces, and institutional contexts. This interpretation is consistent with the International Classification of Functioning, Disability and Health, which defines participation as involvement in life situations and conceptualizes environmental factors as facilitators or barriers to functioning and participation [20,21].

Previous rehabilitation research has emphasized that environmental barriers and supports influence participation across multiple levels, including immediate interpersonal environments, community settings, information access, transportation, service systems, and broader societal structures [22,23]. However, many studies have treated environmental factors primarily as conditions to be assessed or barriers to be identified. The present study adds to this literature by showing how Community Nurses actively modified such environmental conditions in routine practice. CNs reduced psychological barriers by creating low-threshold participation spaces, strengthened relational trust through recognition and validation, enabled residents to assume contributor roles, and bridged digital and institutional barriers through smartphone support and coordination with local administrative structures.

The findings are also consistent with community-based rehabilitation principles. The WHO Community-Based Rehabilitation guidelines conceptualize CBR as a community-based development strategy that promotes inclusion, participation, quality of life, and empowerment [24]. In this study, CNs enacted these principles not through a formal rehabilitation program, but through everyday community-embedded nursing practices. By using familiar community spaces, facilitating resident-to-resident support, and connecting residents with community and municipal resources, CNs helped reconstruct environments in which participation became more feasible.

These findings are also relevant to the social prescribing literature, which emphasizes connecting individuals with non-clinical community resources to address social isolation, loneliness, and participation-related needs [25]. However, CNs’ practices in this study extended beyond referral or linkage. CNs did not simply direct residents to existing resources; they actively shaped the environments into which residents were invited. They prepared accessible spaces, fostered relational safety, supported reciprocal roles, and coordinated digital, community, and institutional resources. This suggests that CNs may complement social prescribing by modifying participation environments themselves, particularly in rural communities where formal services and transportation options may be limited.

By focusing specifically on environmental modification, this study also clarifies the distinct contribution of the present analysis compared with previous research on participation reconstruction and personal-interest-based facilitation within community nursing practice [23,26]. The present study shows that participation was enabled through the active reconstruction of environmental conditions rather than through individual motivation or interpersonal support alone. This contributes to participation-oriented rehabilitation by demonstrating how environmental factors within the ICF framework can be operationalized in real-world rural community practice.

### 4.3. Rural Participation Inequalities and Implications for Practice and Policy

Rural communities often face participation inequalities related to population aging, remoteness, limited transportation, reduced access to services, digital exclusion, and shrinking informal social networks. These conditions can restrict residents’ opportunities to enter community spaces, maintain relationships, access health and welfare resources, and participate in meaningful life situations. The WHO has emphasized that rural health inequities are shaped by adverse social and environmental determinants and weaker health systems in rural and remote areas [27]. Transportation barriers are particularly important for older adults in rural areas, as distance, limited public transportation, and a lack of affordable mobility options can restrict access to healthcare, community activities, and social connections [28].

The significance of this study lies in demonstrating how Community Nurses can address barriers to rural participation through environmental modification. CNs created low-threshold participation spaces, used familiar community locations, offered informal invitations, supported digital communication, and coordinated with local administrative and community organizations. These practices did not eliminate all structural disadvantages associated with rurality, but they created more accessible pathways into participation for residents who might otherwise remain isolated or disconnected.

These findings have practical implications for other rural areas. Rural health and welfare systems should consider supporting community-embedded roles that can work across clinical, social, digital, and institutional boundaries. Accessible community hubs, familiar public spaces, digital inclusion support, and collaboration among healthcare providers, municipalities, welfare agencies, community organizations, and residents may help reduce participation barriers. From a policy perspective, rural community-based care should move beyond service availability alone and consider whether residents can realistically access, enter, and participate in community resources [29]. Evaluations of rural interventions should include participation-oriented outcomes, such as access to community spaces, perceived belonging, reciprocal contribution, digital access, and connection with local resources.

### 4.4. Strengths of the Study

This study has several strengths. First, it provides detailed qualitative evidence on how environmental factors are modified to enable participation, addressing a significant gap in the rehabilitation literature. By focusing on environmental modification processes, this study operationalizes the Environmental factors domain of the ICF framework and provides empirical support for participation-oriented rehabilitation models. In addition, the use of secondary qualitative analysis enabled focused examination of environmental modification mechanisms within an existing longitudinal dataset. Second, the use of longitudinal activity logs and reflective practice records allowed examination of environmental modification processes as they occurred in real-world community settings. These data captured naturally occurring interactions and environmental changes, enhancing ecological validity. Third, this study provides concrete examples of participation emerging through environmental modification, including the creation of participation spaces, relational facilitation, institutional coordination, and resource mobilization. These findings provide actionable insights for healthcare professionals seeking to implement participation-oriented rehabilitation approaches. Finally, the use of thematic analysis informed by the ICF framework enabled systematic and theoretically grounded analysis of environmental modification processes.

### 4.5. Limitations

This study has several limitations. First, the study was conducted in a single rural community, which may limit generalizability to other contexts, including urban settings and different healthcare systems. Environmental modification processes may vary across communities and institutional structures. Second, the study relied on CN activity logs and reflective practice records, which may reflect CN perspectives and may not fully capture residents’ subjective experiences. Future research incorporating interviews with residents could provide additional insights into how environmental modification influences participation. Third, this study focused on qualitative analysis and did not quantitatively measure participation outcomes. Future research integrating qualitative and quantitative approaches may help clarify the relationship between environmental modification and measurable participation outcomes. Fourth, CN activities and environmental modification processes may be influenced by contextual factors such as community culture and healthcare system characteristics, which may affect transferability to other settings. In addition, the records were originally generated for routine practice rather than research purposes; hence, the depth and consistency of description varied across activities and months. In addition, because the study was based on a secondary analysis of routine practice records, two monthly reports were not available in the shared anonymized dataset. This may have affected the continuity of the longitudinal data and limited our ability to capture all environmental modification processes across the year.

## 5. Conclusions

This study demonstrates that CNs enable participation by actively modifying environmental factors within community settings. CNs facilitated participation by creating participation-enabling social environments, strengthening relational environments through trust and validation, restructuring social roles and participation expectations, and bridging digital, structural, and institutional barriers to participation. These environmental modifications enabled participation to emerge through interaction between individuals and their reconstructed environments. These findings contribute to understanding how environmental conditions can be actively modified to support participation-oriented rehabilitation in community settings. Environmental modification represents a key mechanism through which rehabilitation professionals can enable participation in community settings. Integrating environmental modification into rehabilitation practice may enhance participation and improve health and well-being among community-dwelling populations.

## Figures and Tables

**Table 1:** Analytical boundaries between the previous study and the present study.

Aspect	Previous Related Study [15]	Present Study
**Main focus**	**Reconstruction of social participation through Community Nurse practice**	**Modification of environmental factors that enable participation**
Research question	How social participation is reconstructed in community settings	How Community Nurses modify environmental conditions to enable social participation
Theoretical lens	Social participation and relational facilitation	ICF environmental factors component
Unit of analysis	Episodes of participation reconstruction	Episodes of environmental modification
Coding focus	Processes through which residents re-entered or sustained participation	Community Nurse actions that modified relational, social, digital, physical, psychological, or institutional conditions
Data extraction	Segments related to participation reconstruction	Segments specifically related to environmental modification
Contribution	Clarifies how participation emerges through Community Nurse practice	Clarifies environmental modification as a mechanism enabling participation

**Table 2:** Relationship among representative initial codes, candidate subthemes, and final themes.

Final Theme	Number of Coded Segments, n	Candidate Subthemes	Representative Initial Codes
Creating participation-enabling social environments	24	Lowering entry barriers; creating flexible access; making community spaces participation-ready	Creating a drop-in space; allowing observation before participation; adjusting seating and room arrangement; preparing games and recreational activities; keeping Midori no Ie open throughout the day
Strengthening relational environments through trust and validation	20	Building relational safety; validating residents’ identities and interests; expanding interpersonal connections	Listening to residents’ life histories; remembering preferences; validating personal interests; introducing residents with shared interests; encouraging informal conversation
Restructuring social roles and participation expectations	21	Enabling reciprocal support; shifting from recipient to contributor; supporting peer roles	Resident-to-resident teaching; residents helping with smartphone operation; sharing food; transporting equipment; preparing coffee; assisting with event preparation
Bridging digital, structural, and institutional barriers to participation	17	Reducing digital barriers; connecting residents with community systems; coordinating with local organizations	Supporting LINE registration; explaining QR codes and Wi-Fi; helping with smartphone functions; sharing information about local events; coordinating flyers and approval with local administrative structures

**Table 3:** Examples of environmental modification and participation facilitated by Community Nurses.

Setting	Environmental Modification by CNs	Participation Enabled	ICF Environmental Domain
Community space (Midori no Ie)	CNs organized game-based activities and adjusted the available space and equipment, while residents also provided practical support, such as transportation and setup assistance.	Residents played table tennis, observed others, initiated conversations, and engaged in shared social interaction	e1 Products and technology; e3 Support and relationships
Community café	CNs personally invited socially isolated residents and created psychologically accessible participation environments	Residents attended café gatherings, shared tea and food, engaged in conversations, and developed new social relationships	e3 Support and relationships; e4 Attitudes
Community gathering events	CNs facilitated inclusive participation environments where observers were encouraged to join activities	New participants transitioned from passive observation to active participation in conversations and group activities	e3 Support and relationships; e4 Attitudes
Cultural and intellectual activities	CNs facilitated access to books and encouraged residents to engage in reading and discussion	Residents borrowed books, reflected on reading experiences, and engaged in discussions with others	e1 Products and technology; e3 Support and relationships
Community-organized activities	CNs supported collaborative preparation of activities and resource sharing among residents	Residents provided food, assisted with preparation, and assumed active roles in community participation	e3 Support and relationships; e5 Services, systems, and policies
Institutional coordination	CNs coordinated with local organizations and facilitated approval for community café activities	Sustainable participation environments were established, enabling continued community engagement	e5 Services, systems, and policies

**Table 4:** Themes and corresponding ICF environmental factors.

Theme	Description of Environmental Modification	Concrete Example from CN Activities	ICF Environmental Factor Code	ICF Domain Description
Creating participation-enabling social environments	Creating socially and psychologically accessible spaces in which residents can join community activities flexibly and without formal obligation	CNs organized activities at Midori no Ie and other community settings, adjusted participation spaces, and encouraged residents to drop in and engage in games, conversation, and shared activities	e1, e4	Products and technology; Attitudes
Strengthening relational environments through trust and validation	Strengthening interpersonal support, familiarity, and trust to reduce psychological barriers and promote participation	CNs personally invited residents, listened to their experiences, validated their interests, and facilitated interaction among residents, which fostered ongoing engagement in café and community activities	e3, e4	Support and relationships; Attitudes
Restructuring social roles and participation expectations	Enabling residents to shift from passive recipients to active contributors by sharing skills, knowledge, and practical support within activities	In smartphone support sessions and community events, some residents and community members took on peer-support or teaching roles, while others contributed food, equipment, or assistance with activity preparation	e3, e4, e5	Support and relationships; Attitudes; Services, systems, and policies
Bridging digital, structural, and institutional barriers to participation	Connecting residents with community resources, digital tools, and organizational structures that support continued participation	CNs supported access to smartphones and communication tools, shared information about local events, and coordinated with local organizations and administrative structures to facilitate participation opportunities	e1, e5	Products and technology; Services, systems, and policies

## Data Availability

The qualitative data used in this study comprise routine Community Nurse (CN) practice records, including monthly reports and reflective narratives, collected in a small rural community. Because these data contain context-rich descriptions that may permit indirect identification of individuals, they are not publicly available. De-identified excerpts and additional methodological details are available from the corresponding author on reasonable request, subject to approval by the relevant ethics committee and institutional regulations.
